# Assembling a safe and effective toolbox for integrated flea control and plague mitigation: Fipronil experiments with prairie dogs

**DOI:** 10.1371/journal.pone.0272419

**Published:** 2022-08-08

**Authors:** David Eads, Travis Livieri, Tyler Tretten, John Hughes, Nick Kaczor, Emily Halsell, Shaun Grassel, Phillip Dobesh, Eddie Childers, David Lucas, Lauren Noble, Michele Vasquez, Anna Catherine Grady, Dean Biggins

**Affiliations:** 1 U.S. Geological Survey, Fort Collins Science Center, Fort Collins, Colorado, United States of America; 2 Prairie Wildlife Research, Stevens Point, Wisconsin, United States of America; 3 U.S. Fish and Wildlife Service, National Black-Footed Ferret Conservation Center, Carr, Colorado, United States of America; 4 U.S. Fish and Wildlife Service, Colorado Front Range National Wildlife Refuge Complex, Arvada, Colorado, United States of America; 5 Lower Brule Sioux Tribe, Lower Brule, South Dakota, United States of America; 6 U.S. Forest Service, Wall Ranger District, Wall, South Dakota, United States of America; 7 National Park Service, Badlands National Park, Rapid City, South Dakota, United States of America; University of Pretoria, SOUTH AFRICA

## Abstract

**Background:**

Plague, a widely distributed zoonotic disease of mammalian hosts and flea vectors, poses a significant risk to ecosystems throughout much of Earth. Conservation biologists use insecticides for flea control and plague mitigation. Here, we evaluate the use of an insecticide grain bait, laced with 0.005% fipronil (FIP) by weight, with black-tailed prairie dogs (BTPDs, *Cynomys ludovicianus*). We consider safety measures, flea control, BTPD body condition, BTPD survival, efficacy of plague mitigation, and the speed of FIP grain application vs. infusing BTPD burrows with insecticide dusts. We also explore conservation implications for endangered black-footed ferrets (*Mustela nigripes*), which are specialized predators of *Cynomys*.

**Principal findings:**

During 5- and 10-day laboratory trials in Colorado, USA, 2016–2017, FIP grain had no detectable acute toxic effect on 20 BTPDs that readily consumed the grain. During field experiments in South Dakota, USA, 2016–2020, FIP grain suppressed fleas on BTPDs for at least 12 months and up to 24 months in many cases; short-term flea control on a few sites was poor for unknown reasons. In an area of South Dakota where plague circulation appeared low or absent, FIP grain had no detectable effect, positive or negative, on BTPD survival. Experimental results suggest FIP grain may have improved BTPD body condition (mass:foot) and reproduction (juveniles:adults). During a 2019 plague epizootic in Colorado, BTPDs on 238 ha habitat were protected by FIP grain, whereas BTPDs were nearly eliminated on non-treated habitat. Applications of FIP grain were 2–4 times faster than dusting BTPD burrows.

**Significance:**

Deltamethrin dust is the most commonly used insecticide for plague mitigation on *Cynomys* colonies. Fleas on BTPD colonies exhibit the ability to evolve resistance to deltamethrin after repeated annual treatments. Thus, more tools are needed. Accumulating data show orally-delivered FIP is safe and usually effective for flea control with BTPDs, though potential acute toxic effects cannot be ruled out. With continued study and refinement, FIP might be used in rotation with, or even replace deltamethrin, and serve an important role in *Cynomys* and black-footed ferret conservation. More broadly, our stepwise approach to research on FIP may function as a template or guide for evaluations of insecticides in the context of wildlife conservation.

## Introduction

Emerging and re-emerging diseases pose significant risks to human and wildlife health throughout much of Earth. In this article, we concentrate on plague, a widely distributed zoonotic disease of mammalian hosts and flea vectors. The plague bacterium, *Yersinia pestis*, is perhaps best known for causing the Black Death in 14^th^ century Europe. However, the pathogen associates mostly with free-living rodents, many of which are highly susceptible [1]. Plague also reduces populations of mammals that play little to no role in disease maintenance but are nonetheless susceptible, such as humans and a variety of lagomorphs and carnivores [1]. In its introduced ranges, including North America, plague has cascading effects within ecosystems [2, 3]. Eradication of plague is difficult to impossible and, to date, there is little evidence of functional *Y*. *pestis* resistance in host populations [4]. Therefore, effective plague mitigation is critical [5].

Conservation practitioners most commonly manage plague using insecticides for flea control [6]. Application methods have included dust or liquid boxes and dispensing tubes with attracting baits, dust infusions into burrows and nests, and the use of edible baits laced with systemic insecticides. Here, we discuss fipronil (hereafter FIP), a broad-spectrum insecticide that has traditionally been used in spray and ‘spot-on’ topical applications [7] but has more recently been incorporated into host-fed baits [8]. Following consumption, FIP is metabolized primarily into fipronil sulfone (hereafter SULF) with both compounds sequestered mostly in host fat stores, released into the bloodstream over time, and eliminated primarily through feces and urine. Adult fleas acquire FIP from host blood; flea larvae develop in host nests and may contact FIP when interacting with (or consuming) host or flea feces, and when scavenging on fipronil-killed fleas [8]. Additional modes of flea exposure to FIP are possible, perhaps for multiple flea life-stages. FIP disrupts the flea central nervous system and blocks GABA_A_ receptors, causing hyperexcitation, paralysis, and death [9–16]. In some cases, flea populations are controlled for up to 12 months or more, likely due to effects of FIP treatments on multiple flea life-stages [16].

In addition to positive effects of FIP treatments, such as long-term flea control and potential prevention of plague transmission, unintended impacts on target and non-target species require careful evaluation. FIP has an affinity to insect GABA_A_ receptors, reducing but not eliminating concerns with negative effects on mammals [17]. In contrast, SULF also kills insects, accumulates quickly in mammals, but is eliminated over longer intervals than FIP and is more active in mammals, especially at high, repeat FIP doses [18]. FIP and SULF toxicity experiments have been conducted mostly with mice, rats, birds, fish, and invertebrates, primarily in laboratories [17, 18]. Some studies documented deleterious effects but “effective doses have not typically been matched to realistic field exposure conditions” [17]. Results from laboratory experiments are often difficult to extrapolate to field approaches with single, annual treatments offering very low doses of FIP. Continued experimentation in laboratories, at expected rates of field exposure, and experiments with animals under natural conditions are needed [19].

Here, we examine the use of FIP with black-tailed prairie dogs (*Cynomys ludovicianus*, hereafter BTPDs), which are colonial, burrowing sciurids in the grasslands of western North America. A variety of insecticides have been tested with BTPDs in laboratory and field settings [20], often in the context of plague mitigation for the purposes of conserving black-footed ferrets (*Mustela nigripes*), which are endangered, specialized predators of *Cynomys*. Currently, deltamethrin is the most widely used tool. When applied in dust formulation to *Cynomys* burrows, deltamethrin is often effective in controlling fleas and mitigating plague for annual periods or longer [21–23]. Even so, fleas on BTPD colonies have shown the ability to evolve resistance to deltamethrin after recurring annual treatments [24], a finding that invigorated new research on additional insecticides for use with *Cynomys*, including FIP [16].

Experiments with bait-delivered FIP and BTPDs started in early 2016 [12] and expanded later the same year [16, 25]. In Colorado, wheat seed grain with 0.005% FIP by weight suppressed fleas on BTPDs for at least 6 weeks [12]. During complementary experiments in South Dakota, FIP grain and newly-developed FIP bait pellets suppressed fleas for at least 12–14 months and up to 24 months in many cases [16, 25, 26]. This degree and duration of flea control might eliminate plague epizootics and protect BTPDs and other wildlife during inter-epizootic periods, as observed with deltamethrin [27], but field research is needed for confirmation. Continued research on efficacy and duration of flea control, along with effects on BTPD body condition, survival, and reproduction would also be beneficial.

Below, we describe evaluations of FIP grain safety with BTPDs in captivity and the wild. We also present a new evaluation of flea control, describe the effectiveness of FIP grain in protecting BTPD against epizootic plague in the wild, and quantify the relative speed of applying deltamethrin dust or FIP grain to BTPD colonies:

1. Before we initiated field experiments with FIP grain, research suggested no negative effects on BTPDs in the wild [12]. Nevertheless, we proceeded cautiously. We conducted safety trials, during which we fed FIP grain to BTPDs in captivity and monitored them for ill effects over 5-d and 10-d periods.
2. After observing no negative effects in captivity, we conducted field experiments on flea control; flea reductions were substantial, lasting 12 months or more in nearly all cases [16, 25, 26]. Concurrently, we evaluated effects of FIP grain on BTPD survival.
3. Because effects of FIP may be subtle, we evaluated effects of FIP grain on BTPD body condition, an important positive predictor of BTPD survival [28, 29].
4. We also evaluated effects of FIP grain on BTPD reproduction; FIP may disrupt endocrine functioning and reproduction [17, 18] but potential positive effects of FIP on BTPD body condition may lead to increased reproduction [28, 29].
5. To improve understanding of flea control with FIP grain, we conducted a new field experiment, comparing flea burdens on BTPDs occupying treated and non-treated habitat.
6. We evaluated the efficacy of FIP grain in preventing epizootic plague among BTPDs in the wild.
7. Lastly, we compare the speed of FIP grain application versus deltamethrin dust application to BTPD burrows.

## Materials and methods

### BTPD safety trials in captivity

In early July 2016, 32 BTPDs were trialed for FIP grain safety at the U.S. Fish and Wildlife Service’s National Black-Footed Ferret Conservation Center (NBFFCC), Carr, Colorado, USA. The BTPDs had been live trapped in Boulder, Colorado, from a colony treated with deltamethrin dust to suppress fleas [27] (DeltaDust^®^ 0.05% deltamethrin, Bayer Environmental Science, North Carolina, USA). Each animal was treated with fluid insecticide upon capture to further ensure no fleas were brought to NBFFCC (Pyranha^®^ 0.55% pyrethrin, 5.50% piperonyl butoxide, 1.10% permethrin; Pyranha Incorporated, Houston, Texas, USA). Half of the BTPDs were adults (born in previous years) and half were juveniles (born in spring of the current year, distinguished by size). A safety trial was conducted from 9–18 July 2016. BTPDs were divided into 4 groups of 8 (by age) and placed in communal bins, each furnished with a nest box and plastic tubing as places of refuge. Each bin had a 1.3 cm layer of pine shavings as bedding material and was treated with DeltaDust^®^ to further ensure flea extermination, and to inhibit flies. The BTPDs had access to 2 water bottles ad libitum. One adult group of 8 and one juvenile group of 8 were provided oat-based grain laced with 0.005% FIP by weight ab libitum (Scimetrics Ltd. Corp., Wellington, Colorado, USA). One adult group of 8 and one juvenile group of 8 were provided non-treated grain ab libitum as baselines. Grain was replenished as needed. Timothy hay (*Phleum pretense*) was provided to the BTPDs as food and bedding material. BTPDs were checked daily for ill effects, including signs of lethargy, lack of appetite, or distress. Feces were monitored and if a batch did not appear normal (i.e., relatively firm and brown) a note was made of its condition.

Another safety trial was completed from 10–14 April 2017. This trial served two purposes: it functioned as an additional safety trial, and we indexed the amount of grain consumed by each BTPD [30]. Six BTPDs were randomly categorized into 3 sets of 2 and were individually assigned unique alphabetic codes for identification (A through F). BTPDs were housed individually with the same furnishings and within the same bins described above. Grain was presented to each BTPD in a food dish near the nest box. Each of the three pairs received a different quantity or variety of grain: pair A/B each received ½ cup (~95 g) of FIP grain, pair C/D each received ¼ cup (~48 g) of FIP grain, and pair E/F each received ½ cup of non-treated grain. The grain was weighed before it was provided to BTPDs on 10 Apr and reweighed every day during the trial while the animals were being checked for possible ill effects.

### Summary of field methods and statistical inference

We analyzed data from planned field experiments on BTPD survival, body condition, and reproduction (during which flea control with fipronil grain was effective for ≥12 months [16, 25, 26]), and a new investigation of flea control with FIP grain. Trapping and sampling of BTPDs at Buffalo Gap National Grassland and Badlands National Park, South Dakota were conducted under IACUC protocol 2015–07 (U.S. Geological Survey, Fort Collins Science Center, Colorado).

**Fig 1:**
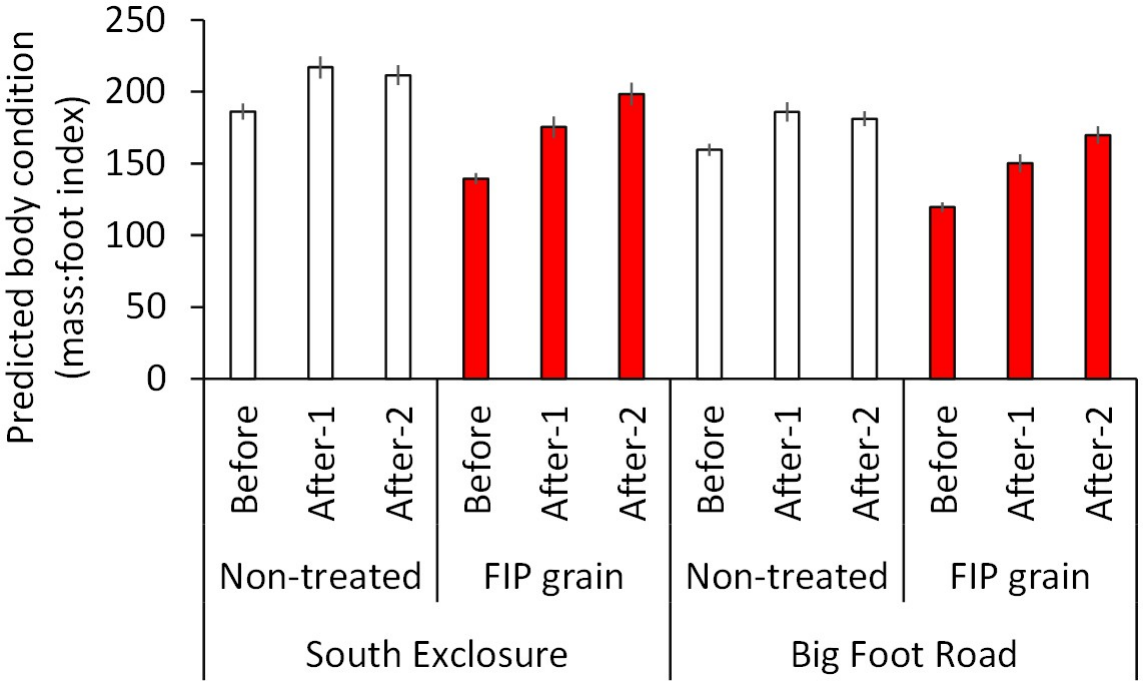
Predicted body condition indices (mass:foot) for black-tailed prairie dogs at South Exclosure and Big Foot Road colonies, South Dakota, USA, 2017. At each colony, data were collected on paired sites treated with 0.005% fipronil (FIP) grain on 24 Jul or left non-treated. Data were collected during 3 BACI (before-after-control-impact) periods: Before FIP grain treatments (June-24 July), After-1 (25 July-30 August), and After-2 (1 September-October). Here, prairie dog ages/sexes are combined for simplicity. Error bars are ± 1 *SE*.

Data were collected in South Dakota, USA, Buffalo Gap National Grassland (43°51’N, 102°03’W), Badlands National Park (43°47’N, 102°08’W), and Lower Brule Indian Reservation (44°03’N, 99°40’W). BTPDs were trapped, aged (adult or juvenile by size), marked with ear-tags, identified, and sampled for fleas under the methods of Eads et al. [16, 25]. At Buffalo Gap and Badlands, sampling occurred on “sites”, defined as collections of juxtaposed burrows that may be treated with FIP grain or remain non-treated as baselines. FIP grain was applied at ½ or ¼ cup per burrow opening on treated sites (Fig 1), which produced similar flea control for at least 12 months at Buffalo Gap and Badlands, 2016–2020 [16, 25]. The new investigation of flea control was conducted at Lower Brule, 2020.

Flea control is a useful measure of the efficacy of tools for plague management. However, a primary metric is the ability of tools to dampen (or even eliminate) plague transmission. A plague epizootic among BTPDs at Rocky Mountain Arsenal National Wildlife Refuge, Colorado (39°48’N, 104°52’W) provided an opportunity to evaluate FIP efficacy in this context, using transects of active burrows to index BTPD population changes on FIP grain treated habitat (½ cup per burrow opening) and non-treated habitat.

Traditional statistical testing approaches (e.g., multivariate models and *P*-values) are valuable with experiments on treatment effects [31]. Below, we describe modeling exercises in which we selected parsimonious models via backward elimination based on *z*-tests for generalized linear models and *F*-tests for analysis of variance (α = 0.050 for single variables and 0.150 for interactions; R x64 version 3.6.1, ‘glmmTMB’ and ‘aov’ [32]). We present point estimates of predictor variables (± 1 *SE*) (‘predict’ [32]). In some cases, we present χ^2^ tests of independence from Microsoft^®^ Excel^®^ (α = 0.050) and simple descriptive statistics.

### BTPD survival in the wild

In the wild, prolonged consumption of FIP grain by BTPDs is seemingly reduced or eliminated unless BTPDs cache the grain, which has not been observed in captivity or in BTPDs generally. Following summer application of FIP grain in the early morning, just prior to periods of high BTPD activity, the grain is usually depleted (or at least not visible aboveground) within about 1 to 6 days [16, 25], although some grain may persist aboveground for much longer, especially in areas with few BTPDs. The concentration of FIP is 0.005% by weight and rodents metabolize and excrete FIP quickly. For example, within 7 days of dosing, rats excrete up to 75% or more of FIP in feces, 6–26% in urine, and 7–18% in bile [33, 34]. With lab rats, the half-lives of FIP and SULF metabolite were estimated to be 8.5 h and 208 h (about 9 days), respectively [35]. SULF is retained longer [33–35] but potential rat mortality from FIP treatment is generally observed within 2 days of dosing [36, 37]. Thus, any negative effect of FIP grain on BTPD survival is expected to be detected within one month of treatment.

We evaluated BTPD monthly survival from July-October 2018 on a colony named South Exclosure, Buffalo Gap. All sites were 1.44 ha in size. Two trapping sites were paired on the northern portion of the colony and 2 were paired on the southern portion. Two sites (1 per pair) were treated with FIP grain (½ cup per burrow) on 23 July and 2 sites (1 per pair) were treated on 5 September. This design allowed for two separate before-after-control-impact (BACI) experiments on monthly survival, with treated sites in the first experiment (July-August) functioning as baseline sites in the second experiment (September-October) and vice versa for the second experiment. Trapping occurred simultaneously on paired sites, and trap densities were set at 31.25 ha^-1^.

We used re-encounters of marked individuals as indices of survival [38]. We ran a binomial model with the following predictor variables: PERIOD (before or after treatment), TREATMENT (not yet treated vs. treated in July, treated in July vs. treated in September), and EXPERIMENT (to evaluate differences between the two BACI experiments). We included all 2-way and 3-way interactions, and PAIR (i.e., experimental plot pairing) as a random effect to link data within pairs. We also included AGE of BTPD, an important predictor of survival [28]. If FIP grain negatively affected BTPD monthly survival, then re-encounters should have been comparatively low from July–August on sites treated in July. Similarly, re-encounter rates might have been comparatively low from September–October on sites treated in September.

We also analyzed data from an investigation of BTPD annual survival (June–October 2018 to June–October 2019). In this case, data came from the 4 South Exclosure FIP sites mentioned above and 3 non-treated sites, each 1.44 ha, on a separate colony (Prairie Wind) at Badlands National Park. Trap densities were standardized at 31.25 ha^-1^. In 2018, trapping occurred nearly simultaneously on treated and non-treated sites. In 2019, precipitation and muddy conditions reduced access to the South Exclosure; thus, we accounted for 2019 trap effort in analyses (TRAP-DAYS, range by site = 20–26 d). We also included variables for AGE and TREATMENT. If FIP negatively affected BTPD annual survival, then re-encounters should have been lower on the 4 FIP sites than the 3 non-treated sites.

### BTPD body condition in the wild

Non-lethal effects of FIP on mammals, including potential positive or negative effects on body condition, may manifest over several months. For example, when FIP suppresses fleas [16, 25] hosts are partly or fully freed of the energetic costs of flea parasitism, allowing for potential increases in BTPD body condition [39–41]. Alternatively, FIP might cause lethargy or illness, reducing BTPD foraging and body condition [17].

We analyzed data from planned BACI investigations of BTPD body condition at paired sites on the same colonies (2017) and treated and non-treated sites on different colonies (2018). The first assessment took place from June–October 2017 on 2 pairs of treated and non-treated sites, one pair at South Exclosure (each site 1.44 ha) and another pair at a colony named Big Foot Road (each site 1.20 ha; treatments at ½ cup per burrow opening on 24 July 2017). The second assessment took place from June–October 2018 on 2 treated sites at South Exclosure (½ cup per burrow opening on 23 July 2018) and 3 non-treated sites at Prairie Wind (each site 1.44 ha).

We used mass:foot ratios as indices of body condition [41]. The distribution of body condition indices was left-skewed. With the 2017 data, we ran a negative binomial model with body condition index (rounded to the nearest integer) as the response and the following predictor variables: AGE and SEX of BTPD (important predictors of body condition [28, 29]), TREATMENT, and PERIOD (Before: June-24 July, After-1: 26 July-30 August, After-2: 1 September-October). We included a 2-way interaction between the latter 2 variables, and a random effect for PAIR. With the 2018 data, we ran a model with AGE, SEX, TREATMENT, and PERIOD (Before: June-22 July, After-1: 25 July-30 August, After-2: 1 September-October) and evaluated an interaction between the latter two variables. If FIP grain negatively affected BTPD body condition indices then, following FIP grain treatments, the indices should have declined disproportionately more on FIP-treated sites compared to changes on non-treated sites.

### BTPD reproduction in the wild

We consider our investigation of FIP effects on BTPD reproduction a pilot experiment. Adult BTPDs (born in previous years) breed, whereas juveniles do not [28]. In South Dakota, the BTPD breeding season begins in mid- to late-February [28]. In mid-February 2020 (15–16 and 21–22 February), contractors treated a BTPD colony in Badlands National Park (named Roberts) with FIP grain at ¼ cup per burrow opening (*n* = 34,318). Juveniles began to emerge from burrows in May. From late-June through mid-July 2020, we live-trapped BTPDs on three 1.44 ha sites at Roberts (treated) and three 1.44 ha sites at Prairie Wind (non-treated). We combed 0 fleas from BTPDs at Roberts (43 processing events) versus 48 fleas from BTPDs at non-treated Prairie Wind (28 events), with significant flea control suggesting the February 2020 FIP treatment was effective in allowing adult BTPDs to consume the FIP grain. Here, we index BTPD reproduction as total numbers of juveniles and adults captured (minimum numbers alive) on treated or non-treated sites as ratios (juveniles:adults) [42] and compare the ratios using a χ^2^ test of independence.

To further evaluate BTPD reproduction, in late-June 2020 we collected visual counts of BTPDs on 3 additional 2 ha sites at treated Roberts and 3 additional 2 ha sites at non-treated Prairie Wind (all separate from the trapping sites), with counts completed in the evening, about 3 h to 30 min before diurnal BTPDs descended into burrows for the night (a time period in which BTPDs are usually relatively active above ground). At each site, 5 repeat visual counts were collected via binoculars from a truck (>50 m from site edges), with 10 min between counts. Here, we analyze summed maximum counts (juveniles:adults) on treated and non-treated sites with a χ^2^ test of independence.

### BTPD flea control in the wild

To supplement prior investigations [16, 25, 26], we evaluated the efficacy of flea control with FIP grain treatments on 2 BTPD colonies at Lower Brule, 2020. Two colonies (Charlie and Charlie South) were treated with FIP grain, each separately at ½ or ¼ cup per burrow opening, on 28 May. A non-treated colony (Highway 47) functioned as a baseline. BTPDs were live-trapped and combed for fleas 70 days post-treatment (½ cup), 72 days post-treatment (¼ cup), and 79 days post-treatment (in this latter case on the non-treated colony). We ran a negative binomial model with flea counts from BTPDs as the response and AGE and SEX of BTPD (often important predictors of flea abundance [16]) and TREATMENT as predictor variables.

### FIP prevention of epizootic plague among BTPDs

In mid-July 2019, U.S. Fish and Wildlife Service staff noticed severe declines in BTPD densities in the southwestern portion of Rocky Mountain Arsenal National Wildlife Refuge [43]. Such observations are indicative of epizootic plague among BTPDs, as no other factor (in the absence of shooting, poisoning, extreme drought, or burrow flooding) is known to cause such severe declines [44]. On 24 and 29 July, two BTPD carcasses in testable condition were found. Both tested positive for *Y*. *pestis* via real-time PCR at the Colorado State University Veterinary Diagnostic Laboratory. A third BTPD carcass tested positive in August 2019.

From 2017 through 2019, biologists at the Arsenal had proactively used insecticides for plague mitigation [43]. For purposes herein, in 2018–2019 (December to March) and 2019 (September), about 238 ha of BTPD habitat along the northwestern portion of the Arsenal was treated with FIP grain at ½ cup per burrow opening. Another 10 ha of habitat, on the southwest portion of the Arsenal, was left untreated as an experimental baseline.

Here we analyze data from transects of active BTPD burrows from summer 2018 (before epizootic) and fall 2020 (after epizootic). We concentrate on transects from FIP-treated and non-treated habitat, and only those transects sampled in both years. Transect lengths could increase or decrease depending on habitat expansion or contraction from 2018 to 2020 (Table 1).

**Table 1:** Summary of active burrow transects and indexed black-tailed prairie dog (BTPD) densities in 2018 (before plague epizootic) and 2020 (after epizootic) on non-treated (baseline) and 0.005% fipronil (FIP) grain treated habitat, Rocky Mountain Arsenal National Wildlife Refuge, Colorado.

Year	Treatment	Area (ha)	No. transects	Sum transect length (m)	Range BTPD ha^-1^
**2018** **(before epizootic)**	Non-treated	10	6	859	36.52–86.79
	FIP grain	238	27	18,529	17.02–73.75
**2020** **(after epizootic)**	Non-treated	10*	6	861	0–0
	FIP grain	238	27	18,850**	26.09–63.79

*No observable BTPD activity

**Sum transect length increased from 2018 to 2020 on habitat treated with FIP grain, reflecting an increase in BTPD habitat size on that habitat

Individual transects of north-south orientation were 3 m wide and spaced 120 m apart. If >50% of a burrow was within 1.5 m of the transect center, it was investigated and counted as active if the opening was at least 7 cm wide, the end of the burrow was not visible, and fresh BTPD scat (green, black, or dark brown and not desiccated) or vegetation clippings were found within 0.5 m of the burrow. The length of each 3-m wide transect was known (range = 62–1387 m), allowing calculation of active burrow densities in each transect. Prior research shows, active burrow densities correlate positively with BTPD densities [45, 46]. We converted active burrow densities to indexed BTPD densities ha^-1^ using the conversion equation [45],

$$\text{BTPDs}\;{\text{ha}}^{-1}=\left(\text{no}.\;\text{active}\;\text{burrows}\times 0.179/0.566\right)/\left(\text{transect}\;\text{length}\;\text{m}\times 3\;\text{m}\;\text{width}\right),$$

where 0.179 is the regression coefficient for converting numbers of active burrows to numbers of BTPDs and 0.566 is the observability index for BTPDs during visual counts [45].

We analyzed the transect data using analysis of variance [32]. Each transect was categorized by treatment (Table 1). To analyze changes in BTPD densities from before to after the 2019 plague epizootic, and by treatment, we considered a statistical interaction between YEAR and TREATMENT. We used Tukey post-hoc tests for comparisons of interest (‘TukeyHSD’, α = 0.050).

### Speed of FIP grain and deltamethrin dust application

In September–October 2018, a contractor used all-terrain vehicles (ATVs) and measuring cups to distribute ½ cup of FIP grain at individual BTPD burrows on the South and North Exclosures, Buffalo Gap. Each day, the contractor recorded the start and end time for each employee (*n* = 3) and the number of burrows treated by each employee. We calculated the average number of burrows treated per hour, concentrating on time spent treating burrows (excluding time preparing gear, etc.). We compared those calculations to speeds of deltamethrin dust using prior data [47]; deltamethrin burrow treatments were accomplished using ATVs for travel and battery powered Technicide^™^ machines (San Clemente, California, USA) to infuse each burrow at a target rate of 4–6 g of DeltaDust^®^. We considered annual values for numbers of burrows treated with deltamethrin dust per hour, 2008–2014.

## Results

### BTPD safety trials in captivity

All BTPDs appeared in good health for the duration of the first safety trial. One exception was a small amount of mucousy diarrhea found in the adult treatment nest box, 11 July 2016 (3 days into the trial). Besides this incident, diarrhea was not noticed in any group, and all animals appeared healthy. While diarrhea is not highly prevalent in captively-held BTPDs, it has been occasionally noted in BTPDs that were not provided FIP, especially upon change of diet.

In the second safety trial, adult BTPDs may have consumed 5–63 g of grain (Table 2). BTPDs sometimes spilled the grain, perhaps resulting in overestimates of consumption in some cases. Of note, no BTPDs consumed all of the grain offered. BTPDs appeared in good health for the duration of the trial. On average, the BTPDs may have consumed 10.5 g of grain d^-1^. Regarding the 4 animals provided with FIP grain, BTPDs were indexed to have consumed 35–63 g of grain ($\bar{x}=48\;\text{g}$) or 0.002–0.003 g of FIP ($\bar{x}=0.002\;\text{g}$).

**Table 2:** Daily amounts of grain remaining in food dishes provided to adult black-tailed prairie dogs during a fipronil (FIP) grain safety trial, 10–14 April 2017, along with indexed maximum amounts of grain and FIP consumed (FIP grain = 0.005% FIP by weight).

Treatment	Prairie dog	Grain (g) in food dishes					Maximum amount consumed (g)	
		10-Apr	11-Apr	12-Apr	13-Apr	14-Apr	Grain	FIP
**FIP grain**	A	94	84	71	35	31	63	0.0032
	B	96	90	80	58	42	54	0.0027
	C	48	46	45	22	10	38	0.0019
	D	49	45	38	29	14	35	0.0018
**Non-treated grain**	E	94	89	71	56	36	58	.
	F	91	88	86	86	86	5	.

### BTPD survival in the wild

The investigation of BTPD monthly survival from June-October 2018 included 179 and 242 observations in the first and second BACI experiment, respectively. The following variables were sequentially removed from the model: PERIOD × TREATMENT × EXPERIMENT (*P* = 0.789), PERIOD × TREATMENT (*P* = 0.646, at which point AGE was removed, *P* = 0.284), and TREATMENT × EXPERIMENT (*P* = 0.181). TREATMENT was then removed (*P* = 0.300). Thus, given the data, there was no evidence for a net effect of FIP, negative or positive, on BTPD monthly survival. The PERIOD × EXPERIMENT interaction was highly supported (*P* < 0.001). In the first experiment, BTPD monthly survival increased from before to after treatment on the first pair (0.50 ± 0.17 to 0.79 ± 0.30) and declined from before to after treatment on the second pair (0.70 ± 0.20 to 0.52 ± 0.20). In the second experiment, BTPD monthly survival declined from before to after treatment on the first pair (0.70 ± 0.20 to 0.52 ± 0.20) and remained similar over time on the second pair (0.50 ± 0.17 to 0.52 ± 0.20).

The investigation of BTPD annual survival from 2018–2019 included 441 observations. TRAP-DAYS, TREATMENT, and AGE were sequentially removed from the model (*P* = 0.650, 0.712, 0.272). Thus, given the data, there was no evidence for an effect of FIP on annual survival. Overall, 37% (0.37 ± 0.10) of BTPDs captured in 2018 were recaptured in 2019.

### BTPD body condition in the wild

The investigation of BTPD body condition indices during June-October 2017 included 145 and 225 observations from BACI experiments at South Exclosure and Big Foot Road, respectively. The PERIOD × TREATMENT interaction (*P* = 0.004) and all effects were supported (*P* ≤ 0.009). Adult BTPDs had higher mass:foot indices (179.30 ± 1.01) than juveniles (110.45 ± 1.03). Male BTPDs had higher mass:foot indices (169.64 ± 1.02) than females (157.28 ± 1.02). On the non-treated sites, BTPD body condition increased from Before to After-1 and declined slightly from After-1 to After-2. In contrast, on the FIP grain sites, BTPD body condition increased from Before to After-1 to After-2 (Fig 1). Thus, FIP grain may have improved BTPD body condition.

**Fig 2:**
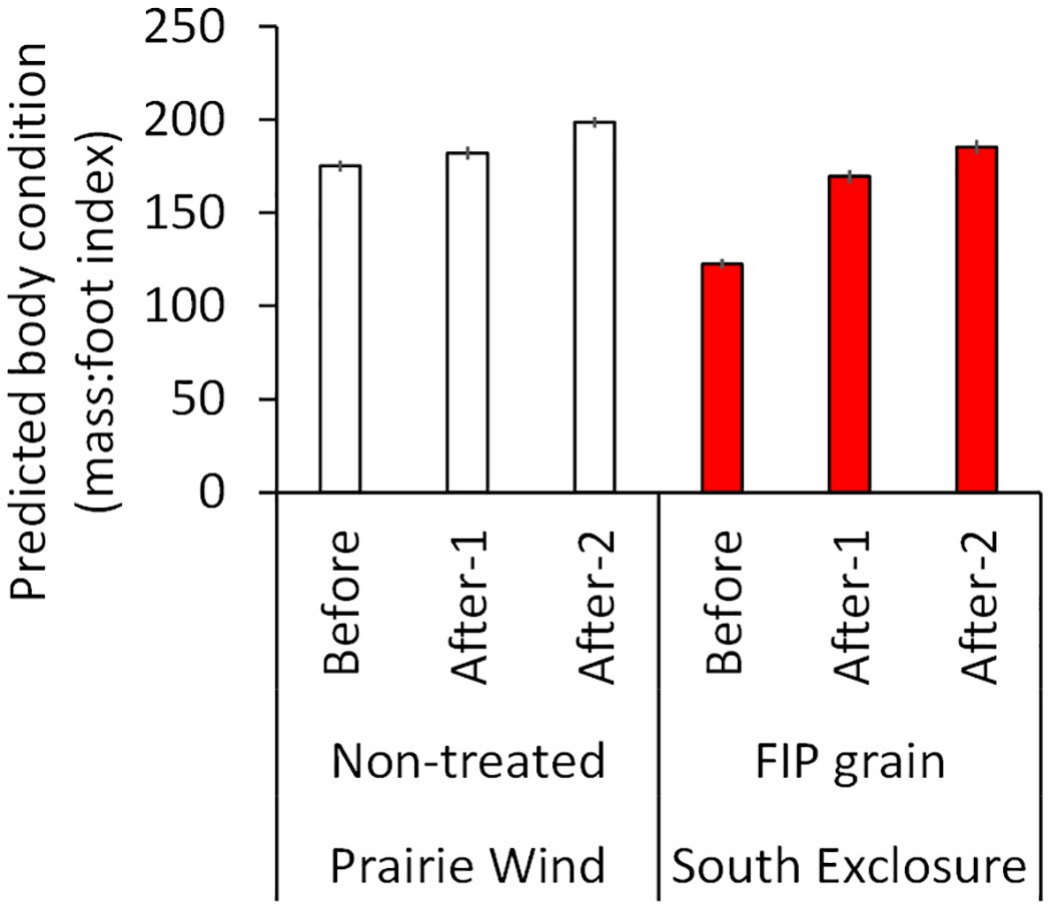
Predicted body condition indices (mass:foot) for black-tailed prairie dogs at Prairie Wind and South Exclosure colonies, South Dakota, USA, 2018. Two sites at the South Exclosure were treated with 0.005% fipronil (FIP) grain on 23 July. Data were collected during 3 BACI (before-after-control-impact) periods: Before FIP grain treatments (June-22 July), After-1 (25 July-30 August), and After-2 (1 September-October). Here, prairie dog ages/sexes are combined for simplicity. Error bars are ± 1 *SE*.

**Fig 3:**
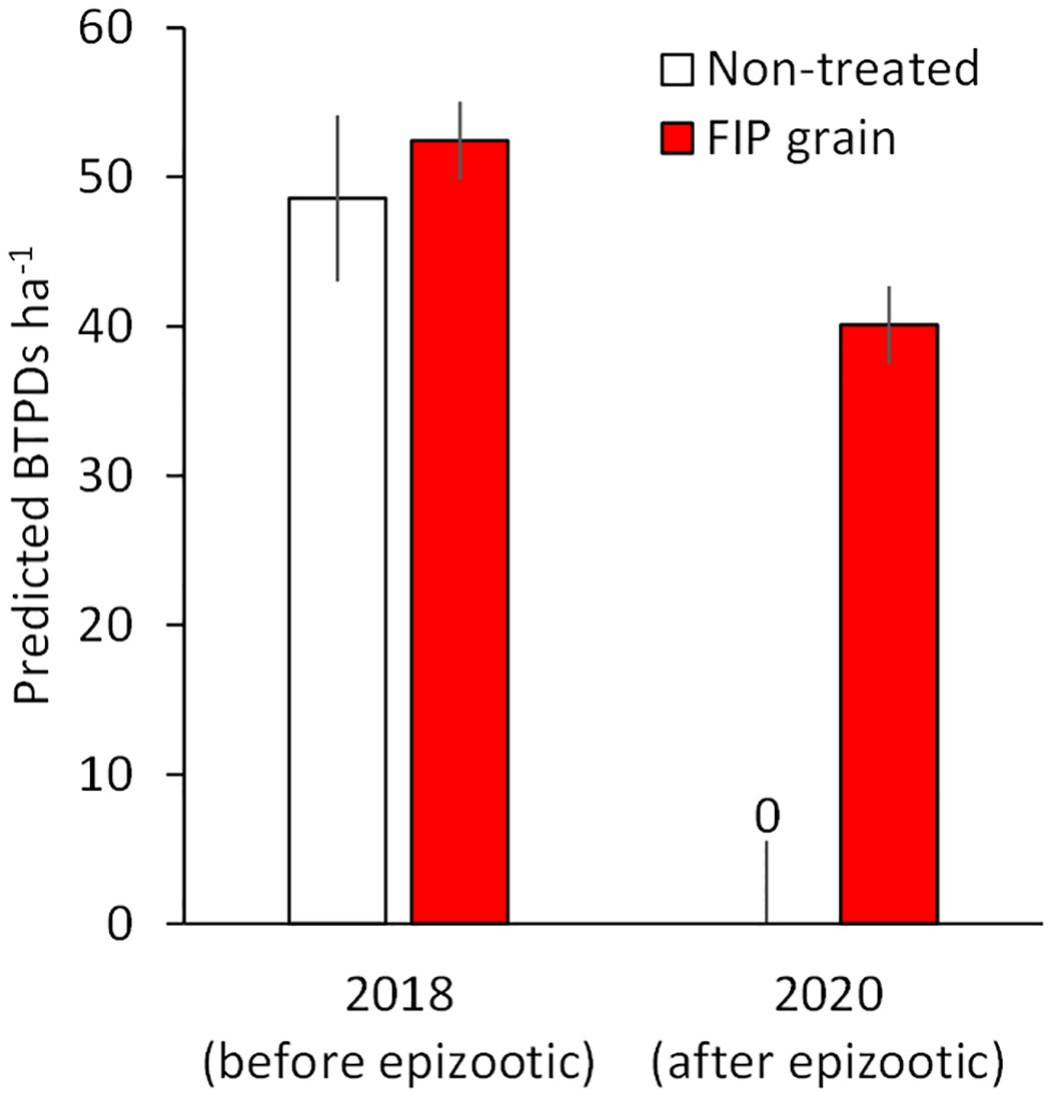
Predicted densities of black-tailed prairie dogs (BTPDs) ha^-1^ in 2018 (before plague epizootic) and 2020 (after epizootic) on habitats with differing plague treatments including non-treated (baseline) and 0.005% fipronil (FIP) grain, Rocky Mountain Arsenal National Wildlife Refuge, Colorado, USA. Error bars are ± 1 *SE*.

The investigation of BTPD condition indices from June-October 2018 included 430 and 572 observations from South Exclosure and Prairie Wind, respectively. The PERIOD × TREATMENT interaction and all effects were supported (*P* < 0.001). Adult BTPDs had higher mass:foot indices (188.85 ± 1.01) than juveniles (105.07 ± 1.02) and male BTPDs had higher indices (177.22 ± 1.01) than females (169.51 ± 1.01). Please note, BTPDs were in better condition in 2018 (more precipitation and vegetative production) than in 2017 (less precipitation); this comparison was not of primary interest and is clouded in Figs 2 and 3 because juvenile BTPDs (smaller than adults) were more abundant in the wetter year of 2018 [for discussion on these topics, see 41]. At both colonies in 2018, BTPD body condition indices increased from Before to After-1 to After-2. The magnitude of increase was stronger on the FIP sites (Fig 2).

### BTPD reproduction in the wild

During the pilot experiment, 65 BTPDs were captured in late-June through July 2020 (30 juveniles, 35 adults) and a maximum of 163 were visually counted in late-June (83 juveniles, 80 adults). Using trapping data, BTPD reproduction (juveniles:adults) was higher on FIP treated sites (29:11) than non-treated sites (1:24) (χ^2^ *P* < 0.001). Using visual count data, reproduction was higher on FIP treated sites (66:37) than non-treated sites (17:43) (χ^2^ *P* < 0.001). Thus, FIP grain may have contributed to an increase in BTPD reproduction.

### BTPD flea control in the wild

The investigation of flea control in 2020 included 37, 26, and 22 observations from sites treated with ½ cup FIP grain per burrow opening, ¼ cup FIP grain per burrow opening, and no cups FIP grain, respectively. SEX (*P* = 0.599), TREATMENT (*P* = 0.288), and AGE (*P* = 0.073) were removed from the model of flea abundance. Fleas were 23% less abundant on the FIP grain sites, but TREATMENT was unsupported as a predictor. On average, fleas were less abundant on the site treated with ¼ cup FIP grain per burrow opening ($\bar{x}=0.50$ fleas per BTPD) than the site treated with ½ cup per burrow opening ($\bar{x}=1.14$ fleas; $\bar{x}$ non-treated site = 1.14 fleas). In contrast to most prior experimental replicates [12, 15, 16, 25, 26], flea control was relatively ineffective.

### FIP prevention of epizootic plague among BTPDs

The YEAR × TREATMENT interaction was highly supported (*P* < 0.001). Roughly 101 ha of non-treated BTPD habitat in the southwest corner of the Refuge suffered almost 100% mortality from epizootic plague in 2019; average BTPD densities along transects in the 10-ha portion of non-treated habitat declined (*P* < 0.001) from 48.57 ha^-1^ (± 5.54) in 2018 to 0 ha^-1^ in 2020 (Fig 3). As the epizootic spread, plague mortality shifted northward along the western edges (non-treated habitat) of the Refuge. The “wave” of plague appeared to have subsided or stopped at BTPD habitat (238 ha) in the northwest portion of the Refuge treated with FIP grain in 2018–2019; on FIP-treated habitat, BTPD densities declined (*P* = 0.008) albeit less so from 52.44 ha^-1^ in 2018 (± 2.61) to 40.10 ha^-1^ (± 2.61) in 2020 (Fig 3).

### Speed of FIP grain and deltamethrin dust application

In 2018, the contractor treated 31,635 burrows with FIP grain over 7 days (25–26 September, 1–3 October, and 25–26 October) during 76.75 work hours, for an application rate of 412 burrows per hour. In a prior report [47], rates of deltamethrin dust application during 2008–2014 ranged from 105 to 199 burrows per hour. Thus, given the data, FIP grain was applied 2 to 4 times faster than deltamethrin dust (for additional comparisons, see [43]). Dusting is slower partly because the applicators must wait several seconds or more for machinery to deposit 4–6 g of dust into each burrow opening (no waiting period is required with FIP grain).

## Discussion

In captivity, FIP grain had no observable effects on 20 BTPDs. During field investigations in South Dakota, we observed and handled hundreds of BTPDs and FIP grain had no observable effect on monthly or annual survival. The acute LD_50_ (the dose required to kill half the treated population) of FIP for Norway rats (*Rattus norvegicus*) is ~97 mg/kg of body mass [17]. Assuming the same LD_50_ for BTPDs, an adult BTPD weighing 916 g (~2 lbs) would need to consume >1.75 kg (>~4 lbs) of 0.005% FIP grain to meet the LD_50_ [17]. In our 5-d safety trial, adult BTPDs consumed 35–63 g of FIP grain, equating to 0.002–0.003 g FIP. Presumably, free-living BTPDs consume comparatively less FIP grain because the bait is (usually) provided once annually [16, 25], not ad libitum, and grain deposited at burrows may be consumed by multiple individuals (and other wildlife). Toxicity is presumably reduced because BTPDs, like all rodents, excrete FIP and SULF; preliminary data suggest elimination may occur within 2–3 to 4–6 weeks, respectively.

In the wild, FIP grain may have contributed to an increase in BTPD body condition. Food supplementation (grain treatment) was brief and minimal, suggesting little direct effect of the grain alone on BTPD condition; in our laboratory study, BTPDs provided with FIP grain and hay consumed a total of 48 g of grain over 5 days, on average, which equates to 0.42% of a BTPD’s indexed annual food intake [48] suggesting little to no effect on BTPD body condition. In other laboratory studies, FIP and metabolites, including SULF, had negligible to negative effects on rodent mass [37], suggesting our results do not reflect direct effects of these compounds. Instead, positive effects of FIP on BTPD condition may relate to reduced flea parasitism. Even at low dosing, FIP treatments usually suppress BTPD fleas considerably, likely due to effects on multiple flea life-stages [14–16, 25, 26], perhaps reducing BTPD stress and energetic requirements and the need for grooming [41], thereby providing BTPDs with more time, and perhaps more motivation [40], for foraging, thus increasing their body condition [41]. Positive effects of FIP on BTPD body condition, if common, may facilitate flea control due to a potential positive feedback loop. Heavier BTPDs are perhaps better equipped for ectoparasite defense with grooming and immune defenses [41] and effective host defenses cause significant flea mortality [49], allowing for cumulative increases in BTPD condition and continued declines in flea densities [50] and so forth, with potential wildlife conservation benefits. We do caution, however, that increased replication is needed to confirm positive effects of FIP grain on BTPD condition. Unfortunately, in this study, BTPDs at the baseline non-treated sites of each experimental pair had higher body condition than those at FIP grain sites at the beginning of the experiments. The greater increase in body condition on the FIP grain sites may simply be the result of lower starting points on those sites.

Although FIP grain might have increased BTPD condition indices, we failed to detect an effect on monthly or annual survival. Perhaps BTPDs were already in adequate condition for reproduction regardless of treatment or increases in BTPD condition were biologically insignificant from the perspective of BTPD survival. Effective flea control on the treated sites [16, 25, 26] would have presumably suppressed or eliminated most plague transmission [27, 50]. A positive effect of FIP on BTPD survival would be expected if plague had been spreading at moderate or high levels at our study sites [27, 51]; existing information from rodents and black-footed ferrets suggest plague transmission was subdued, or perhaps absent, during this particular study.

In Montana, where plague appears to spread at much higher levels [51], Biggins et al. [27] detected a significant, positive effect of deltamethrin flea control on BTPD survival in the absence of epizootics. Biggins et al. suggested plague circulation at lower, enzootic levels was sufficient to cause the positive effect of deltamethrin on BTPD survival. Alternatively, one might suggest flea control increased BTPD body condition, thereby increasing BTPD survival. Our results do not support this supposition. Results from Biggins et al. may indeed illustrate effects of plague on BTPDs (and other *Cynomys*) at sub-epizootic levels, a notion supported by research involving vaccination of sympatric black-footed ferrets against plague [51].

FIP may have increased BTPD reproduction (for similar examples see [52, 53]), perhaps due to positive effects of FIP on BTPD body condition. In studies of laboratory rodents, FIP (fed by gavage at concentrations far exceeding dosages expected for wild BTPDs) sometimes negatively affected female endocrine functioning and altered male sperm quality/quantity, suggesting negative effects on reproduction [17, 18] not found herein with BTPDs. That said, our investigation of reproduction was limited to a single field season, and replication is needed. Most FIP grain treatments are expected to take place in late summer to fall, as vegetation cures, which may increase bait uptake by BTPDs; the timing of such treatments would presumably have little to no direct influence on BTPD reproduction (though indirect, positive influences of flea reductions over annual to biannual periods, and increased BTPD body condition, may influence reproductive output).

**Fig 4:**
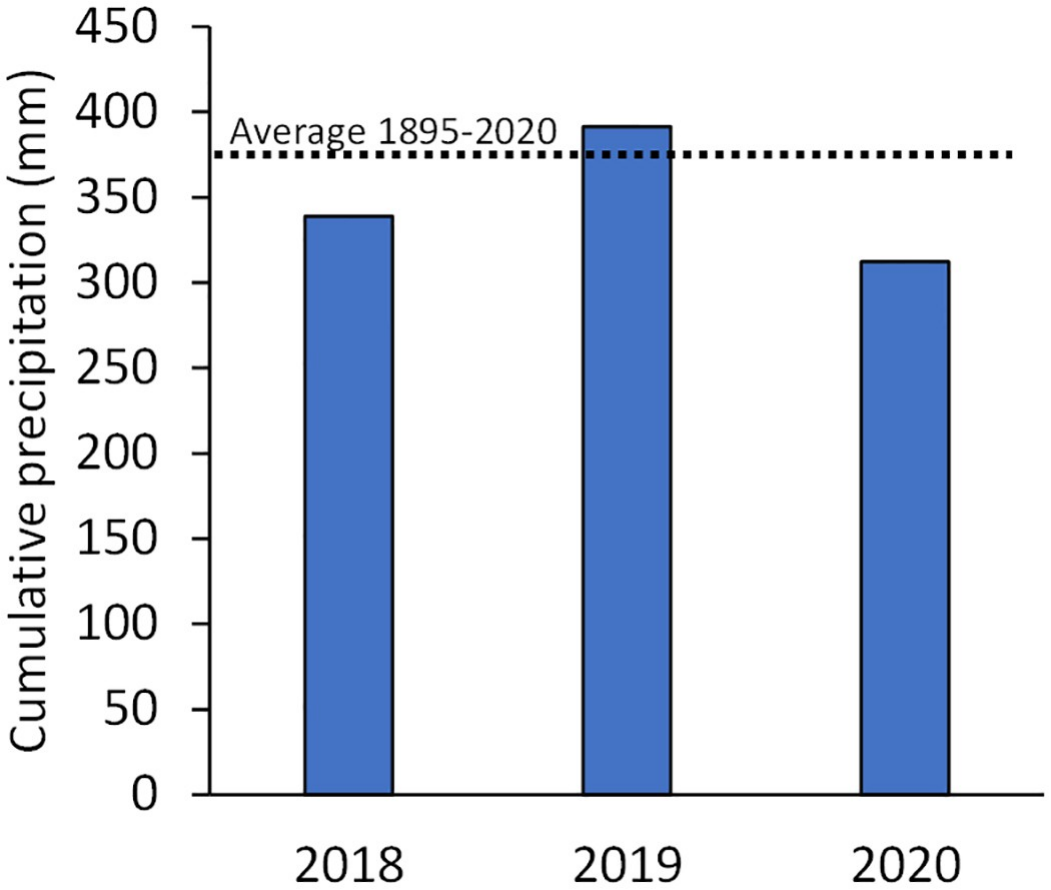
Cumulative precipitation (mm) at Rocky Mountain Arsenal National Wildlife Refuge, Colorado, USA, 2018–2020. The historic average, from 1895–2020 (https://www.prism.oregonstate.edu/), is depicted as a dashed line for reference. Also worthy of note, winter weather was considered extreme in November and December 2019, when an Arctic airmass brought frigid temperatures and snowfall.

Before conducting our FIP grain treatments in areas of suspected or known black-footed ferret occupancy, we conducted safety trials with BTPDs in captivity, detecting few ill effects (results herein). Initial safety evaluations with black-footed ferrets also appear promising. Data analyzed herein, from a ferret reintroduction site in Colorado, suggest FIP grain is effective in preventing epizootic plague among BTPDs; to our knowledge, the first illustration of FIP’s ability to operationally mitigate plague under natural conditions. Epizootic plague and perhaps interacting factors such as weather [44] reduced BTPD densities on non-treated habitat by 100%, versus a 24% decline on habitat treated with FIP grain. Declines in BTPD densities on non-treated habitat can be attributed mostly to plague. The comparatively weaker decline in BTPD densities on FIP-treated habitat may, at least partly, reflect effects of weather on BTPD reproduction and survival, including extreme weather in November and December 2019, and reduced precipitation in 2020 (84% of historic average; Fig 4) [41, 42]. Sub-epizootic effects of plague on BTPDs occupying FIP-treated habitat cannot be ruled out, but any such effects were comparatively (much) weaker than observed on non-treated habitat. Indeed, and generally speaking, in the face of epizootic plague in Colorado, FIP grain maintained BTPDs and, therefore, habitat for black-footed ferrets and their “families” (each with 1 adult female with 3.3 young, and 0.5 adult males [45]). Comparatively, and when converting BTPD densities to numbers of black-footed ferret families [45], the 10 ha of non-treated habitat would have supported ~1 family in 2018 and 0 families in 2020, versus 16 and 13 ferret families on 238 ha of FIP treated habitat. Ferrets have persisted and produced litters on Colorado habitat (and South Dakota habitat) treated with FIP grain.

We caution, while flea control was mostly effective during investigations of FIP grain treatments on BTPD colonies [12, 15, 16, 25], results herein from Lower Brule, with late-May treatments, demonstrate that flea control with FIP grain can be variable. Perhaps the late-May period, when vegetation is growing and sometimes highly nutritious, is an inopportune time to complete FIP grain treatments. No plague mitigation tool is perfect, and we cannot rule out potential negative effects of FIP treatments on BTPDs, black-footed ferrets or other species. From a conservation perspective, it seems net effects are of primary interest. Results herein suggest the net effects are positive, but continued study is encouraged and underway [e.g., 54].

### Recommendations for wildlife managers

Plague is widespread throughout much of the BTPD range and is capable of persisting in areas of disease maintenance, perhaps indefinitely, especially in the “core” of plague’s invaded range [55, 56]. Effective plague mitigation is critical. Deltamethrin and FIP (and other insecticides) might be rotated over time to reduce insecticide resistance among fleas [24]. In some cases on BTPD colonies in South Dakota, deltamethrin resistance developed among fleas after 6–10 y of consecutive annual treatments [24]. Deltamethrin treatments were halted on some colonies; after 2 years, fleas on those colonies were again highly susceptible to deltamethrin, with implications for rotating deltamethrin and FIP over time. When applying deltamethrin dust, ≥4 g could be infused into each burrow; previous research suggests 6–8 g is perhaps better than 4 g [23]. When applying FIP grain, ½ cup [15, 16] or ¼ cup [25] could be distributed near each BTPD burrow opening, regardless of burrow activity. Effective flea control with FIP grain necessitates bait consumption by BTPDs. In sunlight, FIP photodegrades into FIP desulfinyl [57] which, like SULF, can negatively affect vertebrates; though, like SULF, desulfinyl may contribute to flea control [17, 18]. Currently, it may be beneficial for wildlife managers to distribute FIP grain during periods of high BTPD activity and foraging, and in piles that might protect the interior grain from sunlight. Similar recommendations on BTPD activity apply to FIP pellets (“FipBits”), which have produced levels of flea control like those usually, but not always, observed with FIP grain [26]. The relatively open grassland environments on BTPD colonies may assist in photodegradation and, perhaps, hydrolysis of FIP and SULF, which might reduce unintended impacts on non-target species. Monitoring flea populations and assessing flea control with FIP baits may help wildlife managers determine when/where treatments are ineffective, and if possible, the sites might be re-treated to dampen flea numbers. Future research is needed to identify optimal treatment times (seasons) and to evaluate novel ways of encouraging BTPD uptake of fipronil baits (e.g., by increasing the fat content of baits).

Continued research is needed to further evaluate FIP safety and efficacy with *Cynomys*, black-footed ferrets, and other associated species, and net effects on their populations in the wild. As research continues, “red flags” can be discussed in real-time [54, 58]. Integrated plague management is an operational goal to ensure methods are cost-effective and sustainable, while minimizing adverse environmental, economic, or safety consequences for humans, wildlife, or ecosystems. Our stepwise approach to studying FIP safety and efficacy in grain [16, 25] and pellet formulation [26] may function as a template for future evaluations of insecticides in the contexts of disease-vector control and wildlife conservation.
